# Clinical Effect of Tumor-Specific Total Nutrients in Patients with Adjuvant Chemotherapy After Radical Gastric Cancer Resection: A Randomized Controlled Trial

**DOI:** 10.1007/s12029-024-01029-3

**Published:** 2024-02-11

**Authors:** Xiumei Hua, Yang Liu, Yaqing Zhou

**Affiliations:** 1 Department of Critical Care Medicine, Affiliated Hai’an Hospital of Nantong University, Jiangsu Province, Hai’an County, Nantong City, 226600 China; 2 Department of Clinical Nutrition, Haian Hospital of Traditional Chinese MedicineNanjing University of Chinese MedicineJiangsu Province, Nantong, 226600 China

**Keywords:** Gastric cancer, Postoperative chemotherapy, Tumor-specific total nutrients, Enteral nutrition, Immunity

## Abstract

**Background:**

In this study, we combined adjuvant chemotherapy after radical gastric cancer resection with tumor-specific total nutrient therapy to analyze how it affects the nutritional state and immune function of the patient.

**Method:**

We collected data from 106 patients having undergone adjuvant chemotherapy after radical gastric cancer resection between January 2020 and December 2021. We divided the patients into experimental and control groups (with 53 cases in each group) through single-blinded simple randomization using a random number table and the sealed envelope system. The control group received chemotherapy and the regular nutritional diet at the same time while the experimental group received tumor-specific total nutrients based on the control group. We analyzed the index results for the physical examination, nutritional status, and immune function of the patients in both groups recorded before and after one chemotherapeutic cycle.

**Results:**

The control and experimental group compositions were as follows: 58.5% and 52.8% males with a mean age ± standard deviation of 54.36 ± 12.68 and 55.15 ± 12.32 years, respectively. After one chemotherapeutic cycle and the nutritional intervention, the experimental group displayed better physical examination indicators than the control group concerning the weight (55.8 ± 5.41 vs. 54.8 ± 6.94, *p* = 0.621), body fat mass (13.3 ± 0.88 vs. 13.1 ± 0.91, *p* = 0.253), upper arm circumference (21.9 ± 0.94 vs. 21.2 ± 1.23 cm, *p* = 0.001), triceps skinfold thickness (15.1 ± 1.36 vs. 14.3 ± 1.62 cm, *p* = 0.007), and grip strength (23.0 ± 1.30 vs. 22.3 ± 1.33, *p* = 0.007). In addition, the experimental group yielded better nutritional-status indicators than the control, including albumin (35.2 ± 1.60 vs. 33.7 ± 1.44 g/L, *p* = 0.001), hemoglobin (115.7 ± 9.28 vs. 111.5 ± 10.56 g/L, *p* = 0.032), total protein (63.7 ± 5.85 vs. 60.5 ± 5.27 g/L, *p* = 0.004), transferrin (2.5 ± 0.53 vs. 2.2 ± 0.58 g/L, *p* = 0.007), and immune-function indicators CD4^+^ (32.8 ± 4.82 vs. 28.8 ± 3.76, *p* = 0.001), CD8^+^ (34.1 ± 3.36 vs. 37.2 ± 3.85, *p* = 0.001), CD4/CD8 (1.0 ± 0.28 vs. 0.8 ± 0.34, *p* = 0.001), IgA (2.7 ± 1.43 vs. 4.1 ± 1.47, *p* = 0.001), and IgG (8.8 ± 1.74 vs. 10.9 ± 1.28, *p* = 0.001).

**Conclusion:**

Combined tumor-specific total nutrient and adjuvant chemotherapy application after radical gastric cancer surgery effectively improves the nutritional state and immune function of the patients and could be applied in clinical practice.

## Introduction

Gastric carcinoma is a malignant tumor originating from the epithelium of the gastric mucosa and occurring mainly in middle-aged and elderly individuals. Gastric cancer has a low early diagnosis rate and no specific treatment [1]. The overall 5-year survival rate is mildly good in Japan but only 25% in China [1, 2]. In clinical practice, after excluding relevant contraindications, patients with invasive gastric cancer usually receive radical gastrectomy to improve their quality of life by removing the primary tumor, metastatic lymph nodes, and affected infiltrating tissues [3, 4]. After radical surgery for gastric cancer, adjuvant chemotherapy is usually administered to minimize the recurrence rate. However, chemotherapy can easily cause side effects that affect the patient’s chemotherapy tolerance and response, including gastrointestinal reactions, malnutrition, and immune dysfunction [5, 6]. Therefore, reasonable and symptomatic management of chemotherapy side effects is currently a focus of clinical attention. Research suggests that nutritional support therapy improves patients’ nutritional state, reduces complications, and promotes body recovery. Its use in adjuvant chemotherapy after radical gastrectomy for gastric cancer has received considerable clinical attention [7]. Pharmaceutical companies have recently designed specialized enteral total nutrients rich in various nutrients designed for the metabolic characteristics of patients with malignant tumors. In addition, researchers have developed specialized full-nutrient formula powders for cancer patients to provide them with complete nutrition and improve their immune function [8]. However, clinical research on the application and effectiveness of tumor-specific total nutrients is limited, and no further data supports their efficacy. Therefore, this study focused on evaluating the clinical effectiveness of tumor-specific total nutrients in adjuvant chemotherapy after radical gastrectomy for gastric cancer.

## Materials and Methods

### General Data Collection

**Fig. 1:**
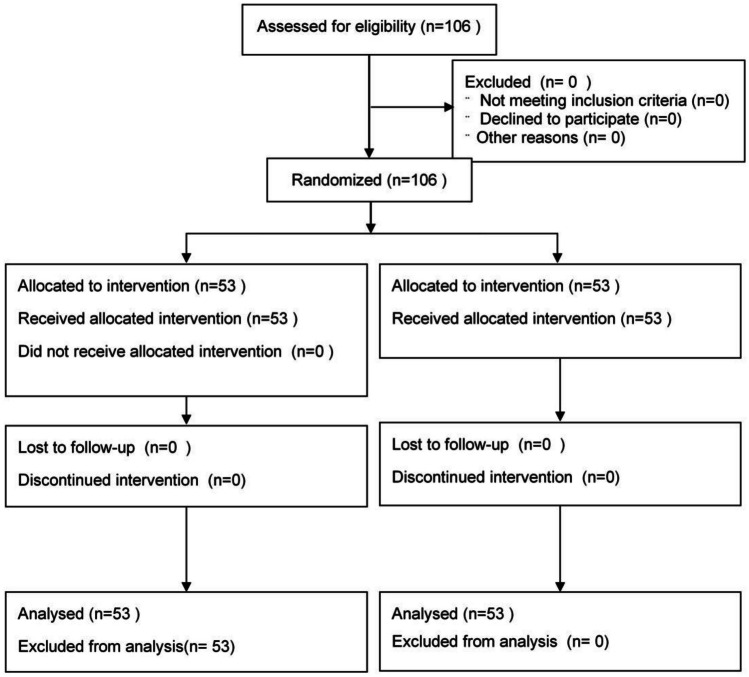
The guidelines’ flow diagram

Clinical data were collected on 106 patients with gastric cancer who met the inclusion criteria after radical surgery at Hai’an People’s Hospital from January 2020 to December 2021. The patients were divided into the experimental and control groups through single-blinded simple randomization using a random number table and the sealed envelope system. Matching was performed for the two groups in terms of general characteristics in accordance with the CONSORT guidelines [9] (the guidelines’ flow diagram is shown in Fig. 1). The primary outcome measure was albumin, and the secondary outcome measures were weight, body fat mass, upper arm circumference, triceps skin fold thickness, grip strength, hemoglobin, total protein, transferrin, CD4+ (%), CD8+ (%), CD4/CD8, IgA, and IgG. The sample size is determined using the sample size estimation formula for comparing the mean of two samples [10]: *n*1 = *n*_2_ = 2*${\left[\frac{\left({t}_{\alpha }+{t}_{\beta }\right)*s}{\delta }\right]}^{2}$, with a 0.05 margin of error, and 95% confidence level, *α* = 0.05, *β* = 0.1, we can obtain from the *t*-value table: *t*_α_ = 1.96, *t*_β_ = 1.28, and our *s* = 1.38, *δ* = 0.9; therefore, $n=2*{\left[\frac{\left(1.96+1.28\right)*1.38}{0.9}\right]}^{2}$ = 49.3. According to the principle of randomized control and single-blindness, they were randomly divided into the experimental group (53 cases) and the control group (53 cases). The data between the two groups were balanced. The study was approved by Medical Ethics Committee of Hai’an People’s Hospital (HKL201937). All patients received chemotherapy and nutritional support after surgery. The control group received postoperative chemotherapy and a conventional nutritional diet; the experimental group received a combination of tumor-specific total nutrients. The follow-up period was 21 days after chemotherapy.

### Inclusion and Exclusion Criteria

The inclusion criteria were (1) age 26–75 years; (2) the diagnosis of gastric cancer was confirmed by pathology, gastroscopy, or related examinations; (3) the gastric cancer TNM stage was II–III, with Billroth II gastrectomy; (4) the patient received standard frontline adjuvant chemotherapy; (5) an expected survival period ≥ 6 months; and (6) the patient and their family members signed an informed consent form to participate in this study.

The exclusion criteria were (1) patients with dysfunction in other important organs, severe infections, various metabolic diseases, and other malignant tumors; (2) tumor recurrence and metastasis that cannot be completely removed; (3) patients with distant metastasis of the tumor; (4) individuals with contraindications to enteral nutrition; (5) patients unable to receive enteral nutrition and diet because of short bowel syndrome or intestinal obstruction; and (6) patients who underwent preoperative chemotherapy or concurrent radiation therapy. In addition, the study also excluded patients who voluntarily withdrew from the intervention process, patients not suitable to continue receiving the intervention because of their own or illness-related reasons, and patients who had poor compliance and could not follow the dietary and nutritional intake plan strictly.

### Methods

#### Surgical and Chemotherapy Methods

All patients underwent laparoscopic Billroth II radical gastrectomy for gastric cancer, and all postoperative patients received a standard first-line adjuvant chemotherapy regimen administered twice daily. After taking the medication for 2 weeks, it was discontinued for 1 week, with a treatment cycle of 21 days.

#### Nutritional Support Methods

The control group received routine nutritional and dietary interventions based on chemotherapy. Within 24 h after admission, the nutritional support group applied the Nutritional Risk Screening 2002 (NRS2002) [11] to assess a patient’s nutritional risk (< 3 indicates no risk; > 3 indicates nutritional risk) and developed a nutritional and dietary plan in accordance with the *Guidelines for Nutritional Treatment of Gastric Cancer Patients* [12]. The patients’ energy demands ranged from 30 to 35 kcal/kg/day, and the total energy distribution throughout the day was 30% breakfast, 40% lunch, and 30% dinner. The daily total calorie allocation is 10–15% for protein, 20–30% for fat, and 50–60% for carbohydrates. Other micronutrients were added following conventional ratios. The patients were provided a routine dietary education and allowed to consume the various nutrients in the diet according to the nutritional support plan.

The experimental group was combined with the control group to apply tumor-specific total nutrients. Within 24 h after admission, the NRS2002 and subjective assessment of patients’ overall nutritional status (PG-SGA) [11] were conducted to develop personalized nutritional support plans. While strictly following the scientific dietary plan, we applied tumor-specific total nutrients designed for the metabolic characteristics of patients with tumors—Enteral Nutritional Emulsion (TPF-T) (Huarui Pharmaceutical Co., Ltd., SFDA approval number H20040722, with a product specification of 200 mL and 300 kcal total calories). Based on a regular breakfast and lunch diet, dinner was replaced with tumor-specific total nutrients; oral nutrients were required to meet at least 30% of a patient’s daily energy needs. This study started from the first day of chemotherapy to the end of the first cycle of chemotherapy.

During the chemotherapy period, both groups of patients were provided with our hospital’s self-made “Dietary Guidelines for the Chemotherapy Period” and “Common Food Nutrient Composition Table.” These materials educate patients and their families on dietary precautions for the chemotherapy period and the importance and requirements of nutritional intervention, aiming to improve their compliance with nutritional support.

### Observation Indicators

The levels of various indicators were compared between the two groups using data taken before and after first cycle of chemotherapy. Physical indicators included body weight, body fat content, upper arm circumference, triceps skin fold thickness, grip strength, and other indicators. Data on nutritional-status indicators were collected using fasting elbow venous blood and an automatic biochemical analyzer and included albumin, hemoglobin, total protein, and transferrin [13]. Data on immune function indicators were collected before and after the nutritional intervention from serum for T lymphocyte CD4^+^, CD8^+^, serum immunoglobulin A (IgA), and G (IgG) levels; these data were used to calculate the ratio of CD4^+^ to CD8^+^ [14].

### Statistical Analysis

All data processing was performed using SPSS 22.0 statistical software. Weight, body fat mass, upper arm circumference, triceps skin fold thickness, grip strength, albumin, hemoglobin, total protein, transferrin, CD4+ (%), CD8+ (%), CD4/CD8, IgA, and IgG levels conforming to normal distribution are expressed as means ± standard errors of the mean; paired samples *t*-tests were used for within-group comparisons, and independent samples *t*-tests were used for between-group comparisons. The *χ*^2^ test was used for comparisons. A *p* value < 0.05 was set to indicate statistical significance.

## Results

### Comparison of Baseline Features

No significant difference was found in baseline features between the two groups of patients (*p* > 0.05) (Table 1).

**Table 1:** Comparison of baseline characteristics of the two groups ($\overline{x} \pm s$ *d*)

Baseline characteristics	Control group (*n* = 53)	Experiment group (*n* = 53)	*χ*^2^/*t*	p
Gender				
Male:female	31:22	28:25	0.344	0.558
Age	54.36 ± 12.68	55.15 ± 12.32	− 0.326	0.745
Weight (kg)	56.4 ± 7.38	56.1 ± 7.15	0.213	0.832
BMI (kg/m^2^)	24.3 ± 3.16	24.2 ± 3.19	0.162	0.872
Tumor sites			0.422	0.810
Gastric antrum	20	21		
Gastric fundus	18	15		
Gastric body	15	17		
TNM stage			0.194	0.907
II	26	25		
III	27	28		

### Comparison of Physical Indicators

The physical indicators of the control group tended to decrease after chemotherapy. No significant difference was found in physical indicators of the experimental group and the control group before chemotherapy. But it showed significant difference after the nutritional intervention in upper arm circumference, triceps skin fold thickness, grip strength between the two groups (*p* < 0.05), but no significant difference was found in weight and body fat mass, indicating that the combination of tumor-specific total nutrients in the experimental group can reduce the negative effect of chemotherapy on some physical indicators (Table 2).

**Table 2:** Comparison of physical indicators between the two groups ($\overline{x} \pm s$ *d*)

Groups		Control (*n* = 53)	Experiment (*n* = 53)	t	p
Weight (kg)	Before	56.4 ± 7.38	56.1 ± 7.15	0.213	0.832
	After	54.8 ± 6.94	55.8 ± 5.41	0.496	0.621
Body fat mass (kg)	Before	13.5 ± 0.84	13.4 ± 0.79	0.631	0.529
	After	13.1 ± 0.91*	13.3 ± 0.88*	1.150	0.253
Upper-arm circumference (cm)	Before	21.8 ± 1.20	22.0 ± 1.37	0.799	0.426
	After	21.2 ± 1.23*	21.9 ± 0.94	3.292	0.001
Triceps skin fold thickness (cm)	Before	16.1 ± 1.58	16.3 ± 1.41	0.688	0.493
	After	14.3 ± 1.62*	15.1 ± 1.36	2.753	0.007
Grip strength (*N*)	Before	23.1 ± 1.28	23.2 ± 1.19	0.417	0.678
	After	22.3 ± 1.33*	23.0 ± 1.30*	2.740	0.007

Compare to the control group, *p < 0.05

### Comparison of Nutritional Status

No significant difference was found in the nutritional evaluation indicators of the experimental group and the control group before chemotherapy. But it also showed significant difference after chemotherapy, including albumin (35.2 ± 1.60 vs. 33.7 ± 1.44 g/L, *p* = 0.001), hemoglobin (115.7 ± 9.28 vs. 111.5 ± 10.56 g/L, *p* = 0.032), total protein (63.7 ± 5.85 vs. 60.5 ± 5.27 g/L, *p* = 0.004), transferring (2.5 ± 0.53 vs. 2.2 ± 0.58 g/L, *p* = 0.007). Therefore, the post-chemotherapy improvement in various nutritional indicators in the experimental group was significantly greater than in the control group (*p* < 0.05) (Table 3).

**Table 3:** Comparison of nutritional status between the two groups (g·L^−1^, $\overline{x} \pm s$ *d*)

Groups		Control (*n* = 53)	Experiment (*n* = 53)	t	p
Albumin	Before	34.2 ± 1.38	34.5 ± 1.54	1.07	0.110
	After	33.7 ± 1.44	35.2 ± 1.60	5.084	0.001
Hemoglobin	Before	113.2 ± 10.83	112.8 ± 11.24	0.187	0.867
	After	111.5 ± 10.56	115.7 ± 9.28	2.175	0.032
Total protein	Before	62.8 ± 5.84	63.0 ± 6.02	0.174	0.925
	After	60.5 ± 5.27*	63.7 ± 5.85	2.959	0.004
Transferrin	Before	2.5 ± 0.64	2.4 ± 0.59	0.836	0.416
	After	2.2 ± 0.58*	2.5 ± 0.53	2.780	0.007

Compare to the control group, *p < 0.05

### Comparison of Immune Function

The experimental and control groups showed no significant difference in immune function indicators on the baselines. After chemotherapy and the nutritional therapy, the experimental group had better immune function indicators than the control group (*p* < 0.05), including CD4^+^ (32.8 ± 4.82 vs. 28.8 ± 3.76, *p* = 0.001), CD8^+^ (34.1 ± 3.36 vs. 37.2 ± 3.85, *p* = 0.001), CD4/CD8 (1.0 ± 0.28 vs. 0.8 ± 0.34, *p* = 0.001), IgA (2.7 ± 1.43 vs. 4.1 ± 1.47, *p* = 0.001), and IgG (8.8 ± 1.74 vs. 10.9 ± 1.28, *p* = 0.001) (Table 4).

**Table 4:** Comparison of immune function between the two groups ($\overline{x} \pm s$ *d*)

Groups		Control (*n* = 53)	Experiment (*n* = 53)	t	p
CD4+ (%)	Before	34.1 ± 4.25	33.7 ± 4.22	0.486	0.645
	After	28.8 ± 3.76*	32.8 ± 4.82*	4.764	0.001
CD8+ (%)	Before	32.6 ± 3.28	33.4 ± 3.23	1.557	0.07
	After	37.2 ± 3.85*	34.1 ± 3.36	4.417	0.001
CD4/CD8	Before	1.1 ± 0.31	1.1 ± 0.29	0	-
	After	0.8 ± 0.34*	1.0 ± 0.28	3.306	0.001
IgA (g·L^−1^)	Before	2.6 ± 1.54	2.5 ± 1.65	0.323	0.718
	After	4.1 ± 1.47*	2.7 ± 1.43	4.970	0.001
IgG (g·L^−1^)	Before	8.4 ± 1.52	8.2 ± 1.45	0.693	0.698
	After	10.9 ± 1.28*	8.8 ± 1.74	7.008	0.001

Compare to the control group, *p < 0.05

### Comparison of Adverse Reactions

No deaths occurred, and no cases were lost during the study period. The vital signs of both patient groups were stable during chemotherapy, and adverse reactions were tolerable. In the experimental group, 23 cases (43.40%) experienced gastrointestinal reactions, including 11 cases of vomiting, 7 of low intake, and 5 of anorexia. In the control group, 37 cases (69.81%) experienced gastrointestinal reactions, including 15 cases of vomiting, 10 of low intake, and 12 of anorexia. The difference between the two groups was significant (*χ*^2^ = 7.528, *p* = 0.006). No significant differences were found in adverse reactions caused by chemotherapy between the two groups, including bone marrow suppression, and the symptoms of adverse reactions were improved after symptomatic treatment.

## Discussion

Research has shown that over half of hospitalized patients with gastric cancer have nutritional risks [15]. A decline in physical fitness affects the activities of daily living and quality of life. Adjuvant chemotherapy after radical gastrectomy for gastric cancer can further control residual cancer cells and reduce the possibility of recurrence; however, it can also easily cause gastrointestinal symptoms, including nausea, vomiting, and decreased appetite, increasing the incidence of malnutrition [15, 16]. Therefore, improving the nutritional support and treatment of patients with gastric cancer is very important.

Patients with gastric cancer should regulate their meal size and frequency, have a balanced diet, and promptly supplement their bodies with nutrients to enhance their immune function, promote chemotherapy effects, and extend their survival [17]. In this study, the control group underwent routine dietary intervention and was supervised to consume easily digestible foods based on their daily calorie intake and energy requirements. Based on the shortcomings of traditional nutritional interventions in postoperative chemotherapy for gastric cancer, this study proposes a specialized total nutrient support therapy for patients with malignant tumors.

Total nutrients include lipids, proteins, dietary fibers, carbohydrates, vitamins, and minerals. The high-energy content in total nutrients can effectively control patients’ weight loss and provide effective energy supplementation even in the event of poor appetite during postoperative chemotherapy. High protein consumption promotes cell and tissue repair. High fat and moderate carbohydrate intake provide energy to patients while reducing tumor growth rate. Dietary fiber can promote intestinal health. The intake of multiple vitamins can improve problems such as loss of appetite and vitamin deficiency caused by chemotherapy. Applying multiple minerals can improve patients’ trace element deficiency and promote an appetite. Tumor-specific total nutrient support therapy can effectively enhance immune function, prevent weight loss and muscle atrophy, improve symptoms like cachexia, and reduce side effects [18].

After one chemotherapy cycle of tumor-specific total nutrient intervention, the physical fitness, nutritional status, and immune function indicators of the experimental group of patients improved compared with the control group. No deaths or serious complications occurred within one cycle of chemotherapy in either group; however, the incidence of gastrointestinal reactions in the experimental group was significantly lower than that in the control group. Tumor-specific total nutrients help maintain the integrity of the intestinal mucosal structure and barrier function, further improving chemotherapy-induced gastrointestinal adverse reactions such as anorexia and vomiting [19]. In addition, one study reported that depression experienced by patients with gastric cancer affects their nutritional state, which might be improved by administering tumor-specific total nutrients orally along with other interventions such as encouragement, company, and psychological counseling [20].

This study had several limitations. First, this study was conducted in a single center; a multi-center study with a large sample size is required to confirm our results. Second, the one-cycle post-chemotherapy follow-up time can be extended to observe further experimental results such as readmission frequency, survival rate, etc.; the optimal type, intensity, and timing of nutritional intervention remain to be explored. In addition, we did not consider the stage of the gastric carcinomas; late-stage cancers are more invasive and have a greater metabolic demand, increased energy metabolism, and lower nutritional state [5].

In summary, applying tumor-specific total nutrients in postoperative adjuvant chemotherapy for patients with gastric cancer can significantly improve malnutrition, enhance immune function, and enhance chemotherapy tolerance and is worthy of clinical application.

## Data Availability

The datasets generated during and/or analyzed during the current study are available from the corresponding author on reasonable request.
